# Supplementary material for: Urban Flora Riches: Unraveling Metabolic Variation Along Altitudinal Gradients in Two Spontaneous Plant Species
Source: Plants (Basel). 2024 Feb 27;13(5):657. doi: 10.3390/plants13050657 (PMC10934943; doi:10.3390/plants13050657)
Supplement: Supplementary file 1 [file plants-13-00657-s001.zip › plants-2844793-supplementary.pdf]

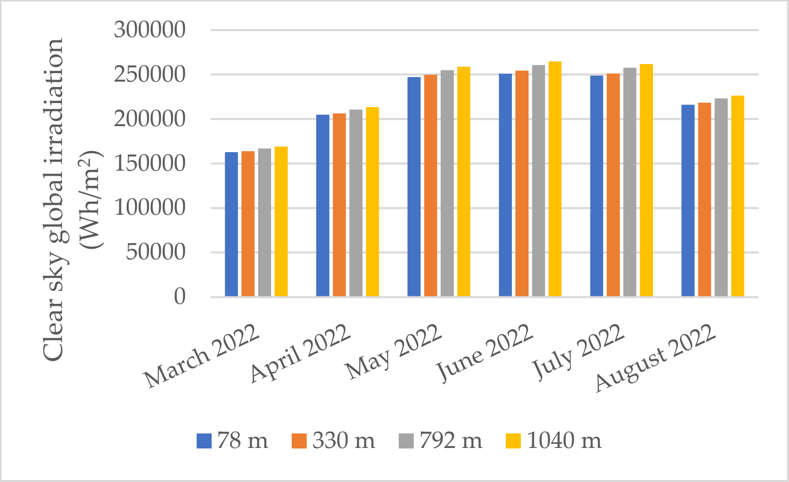

Figure S1. Monthly variation of solar irradiance averages in the four sampling sites.

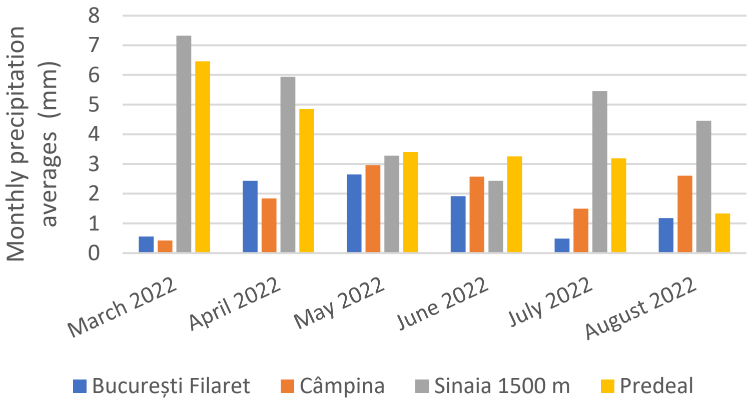

Figure S2. The variation of monthly precipitation averages in the meteorological stations closest to the sampling sites.

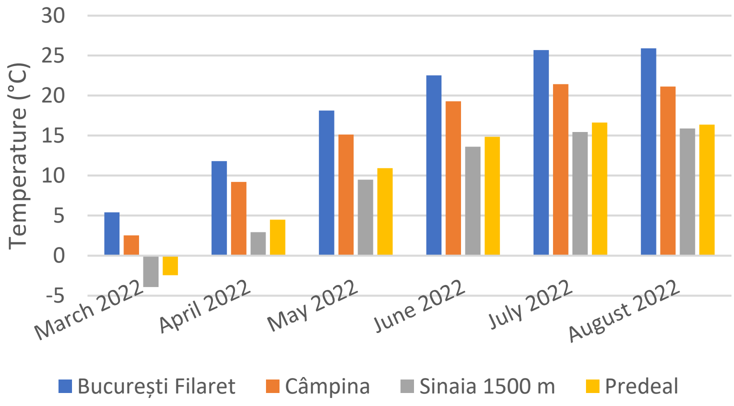

Figure S3. The variation of monthly temperature averages in the meteorological stations closest to the sampling sites.

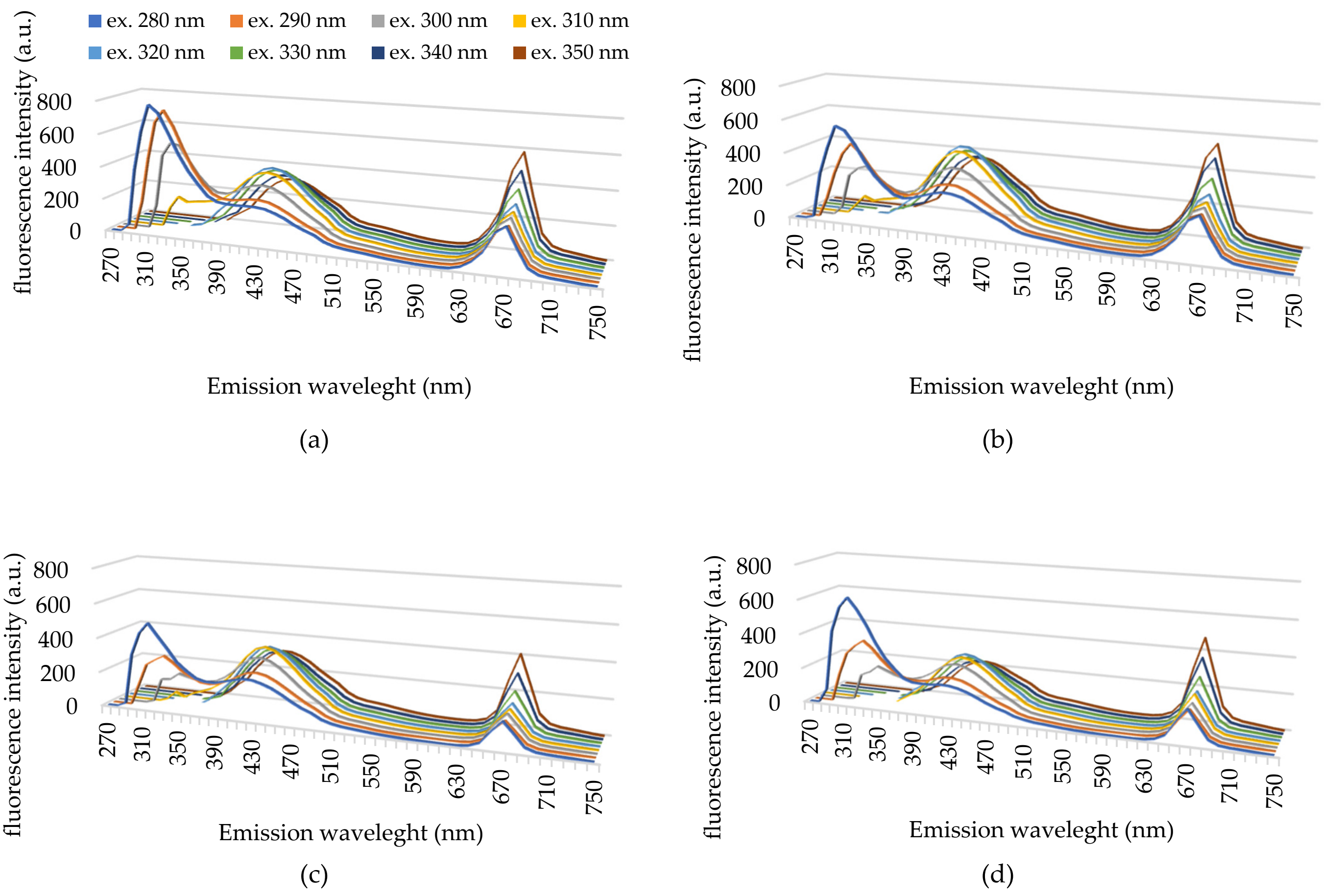

Figure S4. The fluorescence intensity variation of the *L. vulgaris* methanolic extracts excited with UV radiations between 280-350 nm, obtained by leaves collected from the four sites located at 78 m (a), 330 m (b), 792 m (c), and 1040 m altitudes (d).

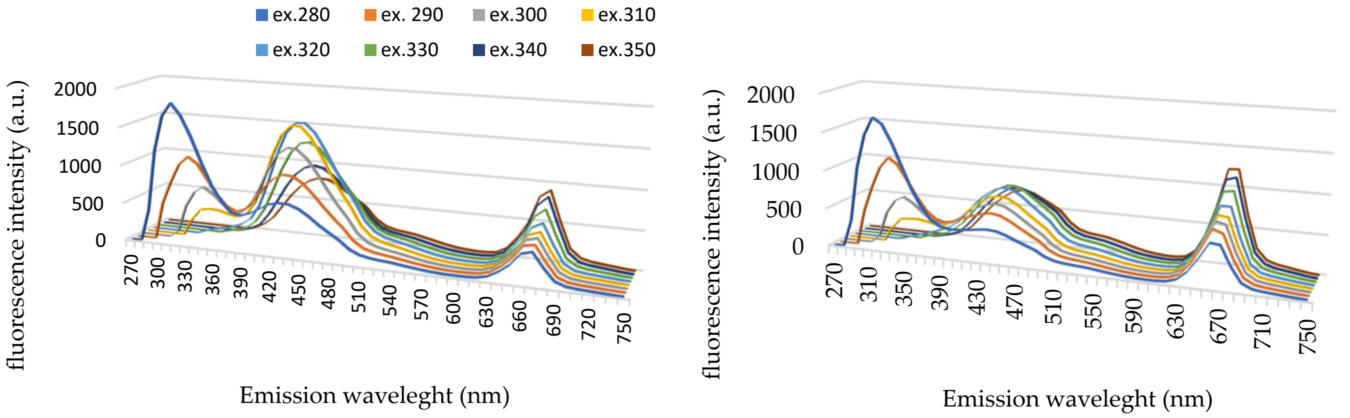

(**a**) (**b**)

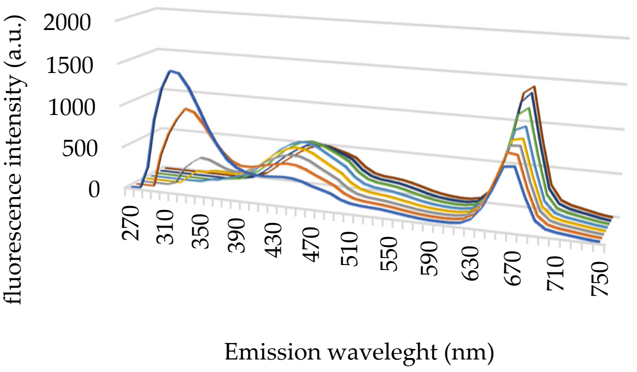

(c)

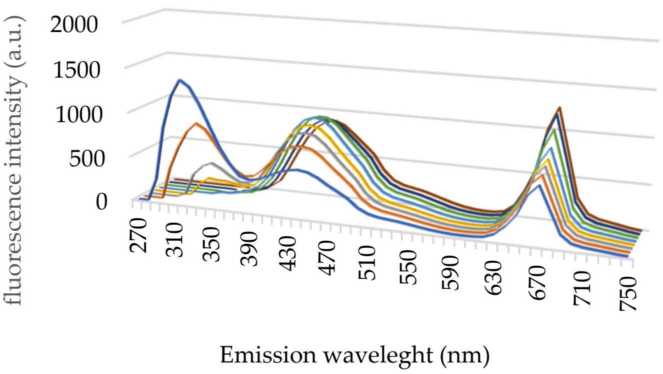

(d)

Figure S5. The fluorescence intensity variation of the *C. intybus* methanolic extracts excited with UV radiations between 280-350 nm, obtained by leaves collected from the four sites located at 78 m (a), 330 m (b), 792 m (c), and 1040 m altitudes (d).

Table S1. The pedological characteristics of the sites.

Site	Soil type
Site 78m	Dark reddish-brown (including Leached chernozems)
Site 330m	Rendzinas (black and brown). Brown soils and rock outcrops
Site 792m	Brown and lessive brown
Site1040m	Acid brown

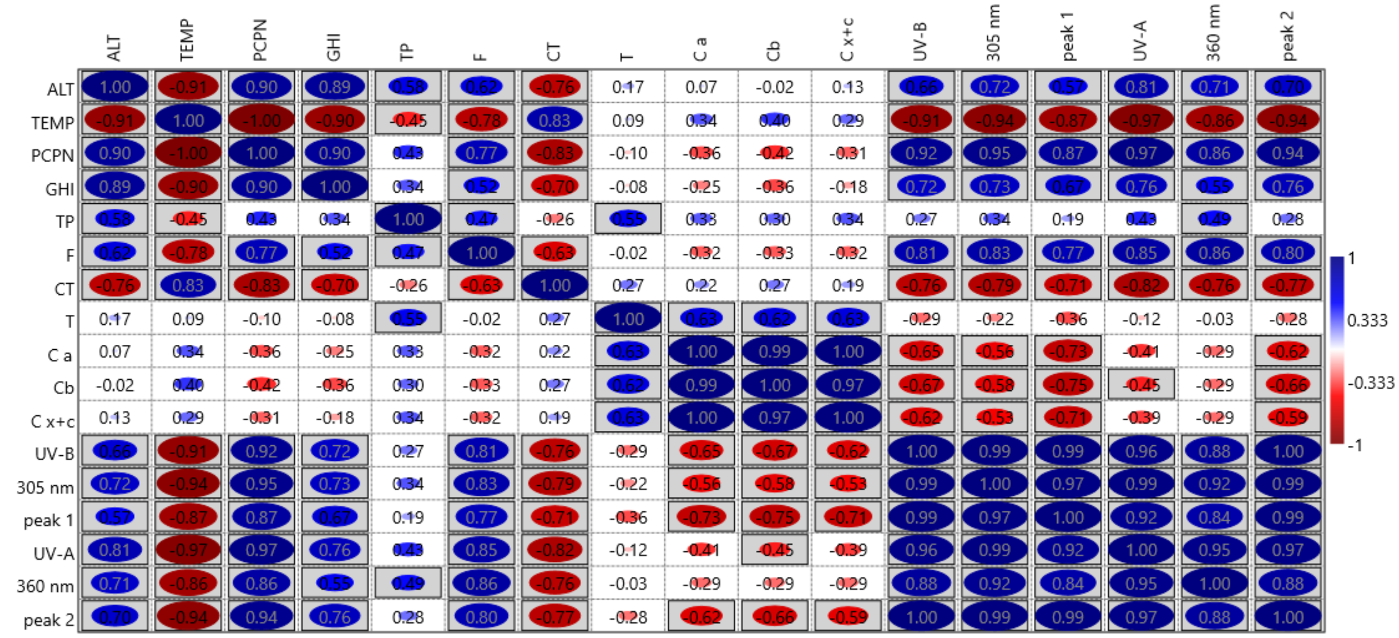

a) *L. vulgaris*

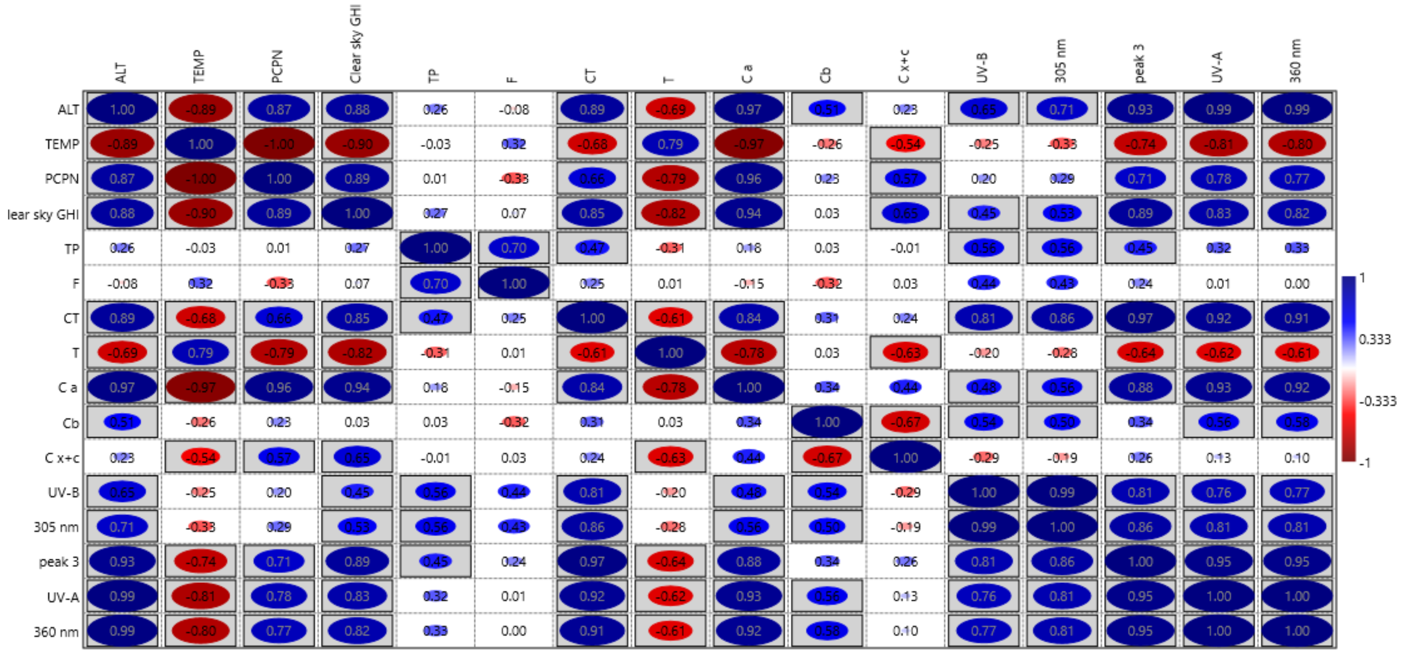

b) *C. intybus*

Figure S6. Pearson's correlation coefficients for *L. vulgaris* (a) and *C. intybus* (b) were expressed as *r* values for the following variables: ALT (altitude), TEMP (average temperature from March to August 2022), PCPN (average precipitation from March to August 2022), GHI (average clear sky global irradiation from March to August 2022), TP (average concentrations of total polyphenols), F (flavonoids), CT (condensed tannins), T (triterpenes), Ca (chlorophyll a), Cb (chlorophyll b), C x+c (carotenoids), UV-B (average absorbances in UV-B range, 280-315 nm), UV-A (average absorbances in UV-A range, 316-400 nm), and peak 1 (283-293 nm), peak 2 (316-325 nm), peak 3 (333-342 nm), and for indicator absorbances at 360 nm and 305 nm, respectively. Positive correlations are denoted by blue oval shapes, while negative correlations are denoted by red. The sizes of oval shapes correspond to variation of *r* values between 0 and 1, respectively 0 and -1. Values within gray boxes are significant at $p < 0.05$. Correlation values are presented in the lower left triangle, and the upper right triangle table displays the two-tailed probabilities.
